# The study of hepatitis B surface antigen and anti-HCV in HIV infected patients

**DOI:** 10.1186/1471-2334-12-S1-P18

**Published:** 2012-05-04

**Authors:** N Girish, T Nagarathnamma, K Saileela, B Sreekanth, D Venkatesha

**Affiliations:** 1 Kamineni Institute of Medical Sciences, Narketpally, Nalgonda, India; 2 Bangalore Medical College, Bangalore, India; 3 Govt. Medical College, Mysore, India

## Background

HIV is known to influence the natural history of infections with HBV and HCV and interactions between HIV and these hepatitis viruses may potentiate HIV replication. The present study deals with the co-infection of HBV and HCV in HIV infected patients and also with the mode of transmission of HBV and / or HCV in HIV infected patients with special reference to high risk groups.

## Methods

The study group included 106 HIV positive patients with history of heterosexual contact and the control group included 30 asymptomatic healthy individuals. The sera of patients were tested for HBsAg and anti-HCV using third generation ELISA kits. The samples positive for first kit were retested for confirmation of results. The sera of control group were simultaneously screened for HIV, HBsAg, and anti-HCV by ELISA method.

## Results

In the study of 106 HIV seropositive patients, 22 (20.75%) were positive for HBsAg and 5 (4.72%) were positive for anti-HCV. One patient (0.94%) was positive for both HBsAg and anti-HCV. Among the 30 healthy controls, one individual (3.3%) was positive for HBsAg and none were positive for HIV and anti-HCV. The co-infection of HIV and HBV in the study population was statistically significant when compared to controls (p<0.05).

## Conclusions

The sexual transmission of both HBV and HCV is of epidemiological importance in the light of heterosexual transmission of HIV in India. Monitoring of HIV infected patients for concurrent infection with HBV and HCV is therefore necessary.

